# Class I MHC Polymorphisms Associated with Type 2 Diabetes in the Mexican Population

**DOI:** 10.3390/genes13050772

**Published:** 2022-04-27

**Authors:** Paola Mendoza-Ramírez, Mildred Alejandra López-Olaiz, Adriana Lizeth Morales-Fernández, María Isabel Flores-Echiveste, Antonio de Jesus Casillas-Navarro, Marco Andrés Pérez-Rodríguez, Felipe de Jesús Orozco-Luna, Celso Cortés-Romero, Laura Yareni Zuñiga, María Guadalupe Sanchez Parada, Luis Daniel Hernandez-Ortega, Arieh Roldán Mercado-Sesma, Raúl C. Baptista-Rosas

**Affiliations:** 1 Facultad de Ciencias Biológicas, Benemérita Universidad Autónoma de Puebla, Puebla 72570, Mexico; paola.mendozaramirez1@gmail.com; 2 Escuela de Nutrición, Universidad del Valle de Atemajac, Zapopan 45037, Mexico; milyolaiz@gmail.com; 3 Escuela de Medicina, Centro Universitario de Tonalá, Universidad de Guadalajara, Tonalá 45425, Mexico; ady15380@gmail.com (A.L.M.-F.); isa970325@gmail.com (M.I.F.-E.); antoniocasillasgdl@gmail.com (A.d.J.C.-N.); lauray.zuniga@academicos.udg.mx (L.Y.Z.); maria.sparada@academicos.udg.mx (M.G.S.P.); 4 Programa de Doctorado en Tecnologías de la Información y Centro de Análisis de Datos y Supercómputo, Universidad de Guadalajara, Zapopan 45100, Mexico; marko4788@gmail.com (M.A.P.-R.); felipe.orozco@academicos.udg.mx (F.d.J.O.-L.); 5 Departamento de Química, Centro Universitario de Ciencias Exactas e Ingenierías, Universidad de Guadalajara, Guadalajara 44430, Mexico; celso.cortes@academicos.udg.mx; 6 Centro de Investigación Multidisciplinaria en Salud, Centro Universitario de Tonalá, Universidad de Guadalajara, Tonalá 45425, Mexico; arieh.mercado@academicos.udg; 7 Departamento de Ciencias Biomédicas, Centro Universitario de Tonalá, Universidad de Guadalajara, Tonalá 45425, Mexico; luis.hortega@academicos.udg.mx; 8 Departamento de Investigación, Centro Universitario UTEG, Guadalajara 44460, Mexico; 9 Departamento de Ciencias de la Salud-Enfermedad como Proceso Individual, Centro Universitario de Tonalá, Universidad de Guadalajara, Tonalá 45425, Mexico; 10 Hospital General de Occidente, Secretaría de Salud Jalisco, Zapopan 45170, Mexico

**Keywords:** HLA, MHC class I, polymorphism, variant, type 2 diabetes, Mexican

## Abstract

Type 2 diabetes (T2D) has been linked to the expression of Human Leukocyte Antigens, principally to the Major Histocompatibility Complex Class II, with only scarce reports of Major Histocompatibility Complex Class I in specific populations. The objective of the present work was to explore the presence of polymorphisms in the MHC Class I related to T2D in the Mexican population using the Genome-Wide Association Studies Slim Initiative in Genomic Medicine of the Americas (GWAS SIGMA) database. This database contains information on 3848 Mexican individuals with T2D and 4366 control individuals from the same population without a clinical or hereditary history of the disease. The searching criteria considered a *p*-value of <0.005 and an odds ratio (OR) of >1.0. Ten novel, statistically significant nucleotide variants were identified: four polymorphisms associated with HLA-A (A*03:01:01:01) and six with HLA-C (C*01:02:01:01). These alleles have a high prevalence in Latin American populations and could potentially be associated with autoimmunity mechanisms related to the development of T2D complications.

## 1. Introduction

Diabetes is defined as a group of metabolic diseases characterized by hyperglycemia resulting from defects in insulin secretion, insulin action, or both. This hyperglycemia is associated with the long-term damage, dysfunction, and failure of various organs such as the eyes, kidneys, nerves, heart, and blood vessels [1].

Specifically, type 2 diabetes (T2D) is a polygenic disease that is caused by a complex interplay between genetic, epigenetic, and environmental factors, with most of the associated genomic regions affecting the regulation of β-cell function focusing on the secretion and resistance of insulin [2,3]. In contrast, Type 1 diabetes (T1D) is defined as a chronic autoimmune disease characterized by insulin deficiency and resultant hyperglycemia due to pancreatic β-cell loss [4].

Previous long-term research has documented that progressive T2D with β-cell deterioration determines the onset and rate of progression, with secondary treatment failure and the need for insulin [5,6] until the A Diabetes Outcome Progression Trial (ADOPT) study showed that the main classes of antidiabetics used in monotherapy resulted in a progressive glycemic increase over time, which may reflect a reduction in insulin secretion [7]. This suggests a progressive disease not related to peripheral glucose resistance and probably associated with autoimmunological damage. It has been proposed that T1D and T2D share some pathophysiological mechanisms; however, the elements involved in this association have not been defined [8,9].

The Major Histocompatibility Complex (MHC), initially defined in humans as the Human Leukocyte Antigen (HLA), is located on chromosome 6p21.3 [10]. MHCs are glycoprotein molecules bound to the cell membrane; they participate in the induction of the specific immune response through the presentation of the antigen to the T lymphocytes [11].

Most experts agree that HLA is the principal region of risk for developing T1D, and less important for T2D. Recent experimental research shows evidence of biochemical consequences of the nonenzymatic reaction of oxidative alterations in key components of MHC in vivo under conditions of hyperglycemia-induced metabolic stress. These modifications were linked to epitope-specific changes in endosomal processing efficiency, MHC-II peptide binding, and editing activity [12]. These findings highlight a link between glycation reactions and altered MHC antigen presentation that may contribute to T2D complications.

Although the correlation of T2D with some MHC-II variants has been more frequently expressed [13,14,15], the association with polymorphic regions in loci A, B, and C is less often mentioned [16]. Genome-wide association studies have linked MHC loci with T2D [17], the killer-cell immunoglobulin-like receptor, and MHC-I interactions that modify the NK cell cytotoxic activity and its production profile. These relations may contribute to the generation of an immune-mediated T2D [18].

The main genetic determinants of T1D are the regions of the MHC, followed by the insulin gene (INS) and the protein tyrosine phosphatase nonreceptor type 22 gene (PTPN22) in chromosomes 6 and 11 [19,20,21]. Some authors have mentioned the association between T2D and histocompatibility antigens, and most of the HLA haplotypes have been associated with a high risk to develop both insulin and non-insulin-dependent diabetes [22,23,24]; most reports have also associated with MHC-II [13,14,15,25,26,27,28,29,30,31].

Based on this evidence, there are several loci not yet classified in or near the HLA complex, which can modulate the risk and evolution of T2D and some complications. Other important evidence: treatments like metformin in T2D improve glycemic levels and delay the onset of chronic complications, and other significant clinical findings through immunomodulation are related to the antigen-presenting function of antigen-presenting cells (APCs). Metformin decreased both MHC-I- and MHC-II-restricted presentation of antigens and suppressed the expression of both MHC molecules and co-stimulatory factors such as CD54, CD80, and CD86 in DCs, but did not affect the phagocytic activity toward exogenous antigens [32].

The Slim Initiative in Genomic Medicine for the Americas (SIGMA) project (https://www.broadinstitute.org/sigma, accessed on 30 October 2020; https://www.broadinstitute.org/diabetes/sigma-t2, accessed on 30 October 2020) set out to systematically identify the genetic risk factors that contribute to this disparity, and translate those findings into improved methods of diabetes treatment and prevention. In the first phase of the project, SIGMA scientists sequenced and characterized more than 10,000 tissue samples from Mexicans and Mexican Americans. This unprecedented analysis led to the discovery of the first identified common genetic variant shown to predispose Latin American populations to the disease. This finding provides unique biological insight into T2D and may present opportunities for therapeutic research and development. Going forward, SIGMA is focused on completing the genetic analysis of T2D in Mexico and translating this knowledge into more effective new approaches to prevention and treatment.

The *GWAS SIGMA* project used Mexican and Latin American populations described in Section 2: the Diabetes in Mexico Study (DMS), the Mexico City Diabetes Study (MCDS), the Multiethnic Cohort (MEC), and the UNAM/INCMNSZ Diabetes Study (UIDS).

To date, there is no published study using the *GWAS SIGMA* database that explains the relationship between MHC-I and T2D. The present work aimed to explore the presence of polymorphisms in the MHC-I related to type 2 diabetes in the Mexican population using the *GWAS SIGMA* database.

## 2. Materials and Methods

### 2.1. Exploration of the SIGMA Database

The *GWAS SIGMA* type 2 diabetes database https://kp4cd.org/sigma is available at http://www.type2diabetesgenetics.org/ (accessed on 30 October 2020). The information was used to explore the frequency of polymorphisms in the Mexican population using the tool variant finder in analysis modules, specifying multiple search criteria to find the genetic variants meeting those criteria. These genomic data were obtained from the principal source described by Williams et al. in 2014 [33].

#### 2.1.1. Diabetes in Mexico Study (DMS)

Individuals enrolled in the study were recruited from two public, tertiary institutions located in Mexico City. The diagnosis of T2D was made based on the American Diabetes Association (ADA) criteria. For our purposes, 811 unrelated healthy subjects older than 45 years and with fasting glucose levels below 100 mg/dL were classified as controls; 569 unrelated individuals, older than 18 years, with either a previous T2D diagnosis or fasting glucose levels above 125 mg/dL, were included as T2D cases.

#### 2.1.2. Mexico City Diabetes Study (MCDS)

A population with diagnostic criteria for type 2 diabetes was recommended by the ADA (fasting glucose of 126 mg/dL or higher or 2 h post 75 g of glucose load 200 or higher). If a participant was diagnosed as diabetic by a physician and was under pharmacologic therapy for diabetes, they were considered diabetic regardless of blood glucose level.

#### 2.1.3. Multiethnic Cohort (MEC)

This group included 2231 T2D cases and 2607 controls, all the Latin American ethnicity.

#### 2.1.4. UNAM/INCMNSZ Diabetes Study (UIDS)

Cases were recruited at the outpatient diabetes clinic of the Department of Endocrinology and Metabolism of a public, third-level, high-specialty medical center. Diagnosis of T2D was made following the ADA criteria, i.e., fasting plasma glucose values of ≥126 mg/dL, current treatment with a hypoglycemic agent, or casual glucose values of ≥200 mg/dL. Control subjects were recruited from a cohort of adults aged 45 years or older, including government employees, blue-collar workers, and subjects seeking attention in medical units for any condition besides those considered as exclusion criteria. Normoglycemic status was defined as having a fasting plasma glucose concentration of <100 mg/dL and no previous history of hyperglycemia, gestational diabetes, or use of metformin.

The pipeline criteria in the *GWAS SIGMA* database were T2D in phenotype or trait; as a dataset, with a *p*-value of <0.005, OR of >1 in HLA-A, HLA-B, and HLA-C loci related to MHC-I. The polymorphisms found were pointed out using the LocusZoom interactive visualization to explore associations of other variants in the HLA region. Linkage disequilibrium (r2) values are based on the 1000G ALL reference panel (including every sample available in the *1000 Genomes* project) (http://www.internationalgenome.org/home, accessed on 2 November 2020) and are supplied by the Michigan Imputation Server (https://imputationserver.sph.umich.edu/, accessed on 7 November 2020).

### 2.2. Analysis and Documentation of Variants Found

The results were tabulated and documented the coordinates of the position in chromosome 6 in GRCh37/hg19 to explore the sequences in UCSC Genome Browser, NCBI dbSNP database number ID, the change in the polymorphisms as a reference/effect allele, the minimum and maximum effect allele average frequency in the general population using the information in *GnomAD* (https://gnomad.broadinstitute.org/, accessed on 2 December 2020), *ExAC* (http://type2diabetesgenetics-old.org/variantSearch/variantSearchWF, accessed on 2 December 2020), which had permission for limited access to the Variant Search database, 1000G (http://www.internationalgenome.org/, accessed on 15 December 2020), *ALSPAC* (http://www.bristol.ac.uk/alspac/, accessed on 4 January 2021), *TWINSUK* (http://twinsuk.ac.uk/, accessed on 11 January 2021) and *GO-ESP* (https://evs.gs.washington.edu/EVS/, accessed on 11 January 2021). The effect allelic frequency and the effect genotype frequency in heterozygous and homozygous were estimated for the Mexican population database using 1000 G available in Ensemble (http://grch37.ensembl.org/index.html, accessed on 1 February 2021); the protein changes were assessed for clinical significance using phenotypes, diseases, or related traits in the ClinVar (https://www.ncbi.nlm.nih.gov/clinvar/, accessed on 22 February 2021) and OMIM databases (https://www.omim.org/, accessed on 26 February 2021); finally, the *p*-value and odds ratio estimated.

### 2.3. IPD-IMGT/HLA Analysis

The polymorphisms found to have statistical significance and odds ratio (OR) greater than 1.0 were identified and used to explore the association with specific HLA alleles in the Immuno Polymorphism (IPD-IMGT/HLA) database, using the polymorphism search tool available at https://www.ebi.ac.uk/ipd/imgt/hla/polymorph.html, accessed on 28 February 2021.

The HLA sequences matches were corroborated by a manual analysis with the following strategy: we obtained the Nucleotide Sequence Data and Genomic Sequence Data for the HLA Allele Report from the IPD-IMGT/HLA database (available at https://www.ebi.ac.uk/ipd/imgt/hla/allele.html, accessed on 1 March 2021), and allele frequencies were evaluated in the Allele Frequencies Worldwide Populations (AFWP) database (http://www.allelefrequencies.net, accessed on 15 March 2021).

### 2.4. Comparison with GEO Dataset

Search matches obtained in the *GWAS SIGMA* T2D database with type HLA-I genetic expression were explored using the Human skeletal muscle–T2D–family history positive individuals’ Mexican American dataset in the Gene Expression Omnibus (GEO) DataSets with accession number GSE21340 [34]. In the same way, HLA-A and HLA-C loci expression were explored and data mining for gene expression analysis was carried out with Orange (https://orange.biolab.si/, accessed on 21 March 2021), using the T2D pancreatic β-cells dataset in GEO with accession number GDS3782 (data is not allowed) [35].

The data were classified into three groups for analysis: a population of Mexican origin with diabetes (Group 1) and their respective controls with positive and negative family history (Groups 2 and 3, respectively). A Mann–Whitney U test was performed to compare the levels of expression in the muscle tissue of the genes identified in HLA-A and HLA-C.

The materials and methodology it is shown as graphic summary in the Flowchart (Supplementary Figure S1).

## 3. Results

### 3.1. HLA Found Variants

From the specified search strategy, 27 variants were found in the HLA-A locus (Table S1), nine variants in the HLA-B locus (Table S2), and six variants in the HLA-C locus (Table S3) related to T2D in a Mexican population with *p* < 0.005.

In the HLA-A locus, only the intronic variants rs72498368 (OR: 1.33), rs199474578 (OR: 1.27), rs707910 (OR: 1.24), and rs2571420 (OR: 1.21) obtained an OR greater than 1.0, and all were associated with A*03:01:01:01 allele; meanwhile, in the HLA-C locus, all intronic variants found, rs17408553, rs2308557, rs1131115, rs2001181, rs1065711, and rs7383157, obtained an OR more than 1.0, and all were associated with HLA-C antigen, Cw-1 α chain precursor (C*01:02:01:01 allele).

The variants found in the HLA-B loci related to T2D in the Mexican population were statistically insignificant, so we decided to focus on the HLA-A and HLA-C variants.

### 3.2. HLA-A*03:01:01:01 Allele

The variants found in HLA-A*03:01:01:01 in T2D in the Mexican population, presented in Table S1, presented specific codon changes different from those reported in the allele database, as shown in Tables S4 and S6. In codons 202 and 68 of the hypervariable peptide-binding region, we identified a difference between the information reported in the *GWAS SIGMA* and IPD-IMGT/HLA databases. For the patient with the dbSNP ID rs199474578, there was no change in threonine, the expected amino acid according to the reading frame, when it is supposed to change to arginine. In the other two cases with OR > 1.0, we found an upstream gene and intron variants without a change in the gene sequence lecture frame that was reported.

### 3.3. HLA-C*01:02:01:01 Allele

In allele C*01:02:01:01, all six variants were found to change the protein sequence and are localized in only three loci in the α chain of HLA-C: rs1065711 and rs2308557 in the locus 101 change serine (S) by asparagine (N) in the protein residue; rs17408553 and rs2001181 in the locus 104 change asparagine (N) by Lysine (K); and rs1131115 and rs7383157 in the locus 123 change serine (S) by tyrosine (Y) in the protein residue (Tables S5 and S6). In all codons, we found a difference between the results reported in the allele database and those reported in the Mexican population with T2D in *GWAS SIGMA*. These polymorphisms, as far as we know, have not previously been reported in the biomedical literature related to T2D.

### 3.4. HLA-C*01:02:01:01 and HLA-C*01:02:01:01 Allelic Frequencies

The allele A*03:01:01:01 has been found in different ethnic groups like African Americans, Arabs, British/Irish, North American, and Northern European Caucasoids; also, in Asiatic Oriental populations from China, this specific allele was reported 11 times and present in 10 populations, as shown in Figure S2. The C*01:02:01:01 was seen in Brazilians Kaingang, British/Irish Caucasoids, Asiatic Indians, and Han and Hong Kong Chinese, Japanese, and Korean Asiatics; this allele was not previously reported in the AFWP database, but a C*01:02:01 frequencies summary is given in Figure S3.

### 3.5. Differential HLA-A and HLA-C Expression Using GEO

The levels of expression in the muscle tissue of the genes identified in HLA-A and HLA-C were compared among three groups for analysis: a population of Mexican origin with diabetes (Group 1) and their respective controls with positive or negative family history (Groups 2 and 3, respectively) (Figure S4). The results were the following:

First, a Kolmogorov–Smirnov test was carried out to determine if the groups had a normal distribution and to confirm that the data were comparable—namely, if they were found in the same range. In this case, the groups could be compared.

A comparison was made between the groups (1 vs. 2, 2 vs. 3, and 1 vs. 3), for this, a Kruskal–Wallis test was performed, in which no significant difference was found.

Then a Mann–Whitney U test was performed to confirm that there were indeed no differences. Group 1 was compared against 2, and no significant difference was found, with the two-tailed comparison equal to 0.594. In the comparison of 1 vs. 3, there was no significant difference (0.391). Group 2 was analyzed against 3, and there was no difference (0.165). In summary, no significant differences were found between the different groups analyzed using the Mann–Whitney U test.

## 4. Discussion

Although several studies have demonstrated that β-cell destruction is related to T1D, there is also evidence of the existence of β-cell autoimmunity in T2D. Hyperglycemia contributes to the increased expression of several β-cell antigens, thereby increasing the vulnerability of β-cells to autoantibodies such as anti-GAD, the most frequently detected autoantibodies in phenotypic T2D. CD8(+) T cells may be cytotoxic upon binding to MHC-I molecules on the surface of pancreatic β-cells [36].

Scarce articles had previously described the potential association between HLA-I and T2D, most of them in specific ethnic groups, like Pima Indian, Finnish, Papua New Guinean, New Zealand Māori, and African American populations. The results of this and other studies show that HLA-A alleles are most frequently related. In the Pima Indians study, HLA-A2 (A*02:01:01:01) and HLA-A24 (A*24:02:01:01) were commonly found, with frequencies of around 49% that match the results in the Finnish population [23].

Moreover, recent studies have demonstrated that an incremental MHC-I gene expression in target tissues may be relevant to the pathogenesis of T2D. Hyperglycemia in non-insulin-dependent patients may increase MHC-I levels in target tissues and contribute to chronic autoimmune complications in advanced disease, leading to specific organ and tissue damage [37], but apparently without functional defects in immune cells, at least in circulating monocytes, dendritic cells, NK cells, and T lymphocytes [38]. However, other analyses demonstrated that CD8(+) and CD4(+) T-cell reactivity to islet-specific antigens in diabetic patients was more prevalent in T1D subjects than in healthy donors, and CD4(+) T-cell autoreactivity appears to be present in both T1D and T2D, while autoreactive CD8(+) T-cells are unique to T1D [39].

Some clinical aspects, such as the early age of initiation of the disease and the rate of β-cell destruction in the large evolution of T2D, could be related to MHC-I [9,40,41]. Previous evidence shows a strong relationship with the HLA-B40 groups of antigens (relative risk 5.1 chi 2 = 16.8, *p* < 0.001); this was mainly attributable to HLA-B48 and HLA-B60 in some specific populations with a high prevalence of T2D [42].

On the other hand, subjects with adult-onset autoimmune diabetes who do not necessitate insulin therapy for at least six months after diagnosis are demarcated as having latent autoimmune diabetes in adults (LADA), a condition more heterogeneous than young-onset autoimmune diabetes that shares clinical and metabolic characteristics with both T2D and T1D. It involves highly variable β-cell destruction, different degrees of insulin resistance, and a heterogeneous titer pattern of islet autoantibody, suggesting that different pathophysiological pathways partially explain the heterogeneous phenotypes of LADA compared to T2D [43,44,45].

Gestational diabetes mellitus (GDM) patients who subsequently developed T2D had a significantly higher frequency of MHC-I locus, specifically HLA-B41 and HLA-Bf-S in the MHC-I locus and HLA-DR2 in the MHC-II locus, and a lower frequency of HLA-DR1 and HLA-DR6 phenotypes in the MHC-II locus than control subjects. Even after controlling for age and body mass index, HLA-B41 and HLA-DR2 were independent predictors of developing insulin-requiring GDM and T2D in GDM subjects. Otherwise, GDM patients who required insulin during pregnancy possessed a significantly higher frequency of HLA-A33 in the MHC-I locus and HLA-DR2, HLA-DR9, and HLA-Bf-S phenotypes in the MHC-II locus than control subjects [46].

The seminal studies related T2D with the increase in the frequency of the MHC-I Cw4 allele with the age of onset, the body mass index, and positive family history [16,47]. Additionally, T2D patients with HLA-B8 and B8/B15 have shown significantly lower C-peptide concentrations (*p* < 0.05) than patients without these HLA antigen polymorphisms. However, subsequent studies using serology have not found an association between T2D and MHC-I (HLA-B or -C) [48].

Furthermore, it is important to consider the possibility that the genetic predisposition in immediate family members with risk of type 1 diabetes caused by the HLA complex could influence the long-term β-cell malfunction in patients with type 2 diabetes [49], which, in turn, could strengthen the idea of a genetic relationship between T1D and T2D and their regulation in the HLA region [14].

In another study on South Indians, HLA-A alleles were also found, but this time not just in a positive significant way; it was found that alleles A*68 (A*68:01:01:01), A*03 (A*03:01:01:01), and A*11 (A*11:01:01:01) showed a significant negative association, which may indicate a protective function [31]. In accordance with previous findings, in a Mexican American study, some HLA-A and -B alleles were associated with T2D protectively; among these were the alleles HLA-A2 (A*02:01:01:01), A25 (A*25:01:01:01), and A3 (A*03:01:01:01), along with some B alleles like HLA-B35 [50].

The association of both groups of alleles, as described previously in three populations, one in South African Indians and two in continental Indians. These variations in the results may indicate that it is a combination of multiple alleles that determine the presence of T2D and that the finding of some alleles does not necessarily determine the risk of developing diabetes; they also might determine the absence or the penetrance of the disease, making it less serious. This is supported by some studies like that by Tuomilehto-Wolf et al. in 1993 [23], wherein the results show the same pattern, and their conclusions were similar.

## 5. Conclusions

Whereas T2D is the most common form of diabetes and non-insulin-dependent in the early stages of the disease, its specific etiology is not yet known; its frequency varies in different population subgroups, and it is often associated with a strong familial, likely genetic, predisposition, more so than T1D. Until now, T2D genetics have been seen as complex and so not clearly defined. Among the subgroup of autoimmune T1D, there is a slowly progressive form of β-cell destruction generally occurring in adults who present with an initial clinical picture of T2D but who have autoantibodies found in T1D and subsequently develop insulin deficiency. Research on this topic is difficult because the HLA loci in chromosome 6 are highly variable and the statistical association of traits and genomic variants requires large groups of individuals to study.

The changes found in our analysis focus on the amino acid residues on the α-1 and α-2 domains, responsible for antigen recognition, which could influence the generation of an immune response against potential antigens not yet defined related to the deterioration of long-term target organs associated with the chronic complications observed in T2D.

Ten novel statistically significant nucleotide variants were identified: four polymorphisms associated with HLA-A (A*03:01:01:01) and six with HLA-C (C*01:02:01:01). These alleles have a high prevalence in Latin American populations and could potentially be associated with autoimmunity mechanisms that participate in the development of type 2 diabetes complications. This relationship could explain some phenomena related to the age of initial presentation of the symptoms, its progression, and the severity of the damage to target organs.

## Data Availability

The datasets used and analyzed during the current study are available from public databases online.

## Supplementary Materials

The following are available online at https://www.mdpi.com/article/10.3390/genes13050772/s1, Table S1: Variants found in HLA-A loci related to type 2 diabetes in the Mexican population with statistical significance *p* < 0.005. Table S2: Variants found in HLA-B loci related to type 2 diabetes in the Mexican population with statistical significance *p* < 0.005. Table S3: Variants found in HLA-C loci related to type 2 diabetes in the Mexican population with statistical significance *p* < 0.005. Table S4: HLA-A*03:01:01:01 protein sequence variants found in a Mexican population with type 2 diabetes. Table S5: HLA-C*01:02:01:01 protein sequence variants found in the Mexican population with type 2 diabetes. Table S6: Polymorphisms found in HLA-A*03:01:01:01 and HLA-C*01:02:01:01 alleles. Figure S1: Methods flowchart. Figure S2: HLA-A*03:01:01:01 allele frequency in worldwide populations. Figure S3: HLA-C*01:02:01:01 frequency in worldwide populations. Figure S4: HLA-A and HLA-C differential expression values from muscle tissue. Figure S5. Fisher’s exact test of the expression values obtained from the group with Type 2 Diabetes (DT2) compared to a control group with a positive family history for DT2 and a control group with a negative family history for diabetes.
