# Supplementary material for: Class I MHC Polymorphisms Associated with Type 2 Diabetes in the Mexican Population
Source: Genes (Basel). 2022 Apr 27;13(5):772. doi: 10.3390/genes13050772 (PMC9140925; doi:10.3390/genes13050772)
Supplement: Supplementary file 1 [file genes-13-00772-s001.zip › genes-1649373-supplementary.pdf]

GCAT TACG GCAT *genes*

MDPI

Table S1. Variants found in HLA-A loci related to type 2 diabetes in mexican population with statistical significance p < 0.005.

Position in chromosome 6 (GRCh37/hg19)	dbSNP ID	Reference/ effect allele	Effect allele average frequency	Effect allelic frequency in Mexican population*	Effect genotype frequency hetero/ homozygote*	Minor allele frequency GWAS SIGMA	Protein change	Consequence	Clinical significance (Other phenotype, diseases or trait related in *ClinVar*)	*P* Value	Odds ratio
29910358	rs1143146	C/G	0.477[5]-0.488[1]	0.547	0.562/0.266	0.348	p.L10V	missense variant	Not Reported	0.000162	0.878
29910970	rs72498368	G/A	0.0280[5]-0.054[1]	0.105	-	**0.0503**		intron variant	Not Reported	0.000242	1.36
29909447	rs9260092	G/C	0.492[1]	0.539	0.578/0.250	0.347		intron variant	Not Reported	0.000257	0.882
29910482	rs9256980	G/A	0.509[#]	0.562	0.375/0.312	0.405		intron variant	Not Reported	0.000272	0.881
29909840	rs2734904	G/A	0.482[5]-0.497[1]	0.586	0.484/0.344	0.343		5' UTR variant	Not Reported	0.000297	0.883
29909559	rs9260101	G/A	0.451[4]-0.491[1]	0.539	0.578/0.250	0.349		intron variant	Not Reported	0.000304	0.883
29909372	rs9260086	C/G	0.476[4]-0.489[1]	0.539	0.578/0.250	0.347		intron variant	Not Reported	0.000310	0.883
29909523	rs9260098	A/C	0.414[3]-0.430[1]	0.586	0.547/0.312	0.374		intron variant	Not Reported	0.000311	0.882

29910429	rs9260123	C/G	0.468[6]-0.495[1]	0.618	-	0.360		intron variant	Not Reported	0.000492	0.886
29909339	rs9260084	G/A	0.472[4]-0.488[1]	0.566	-	0.378		intron variant	Not Reported	0.000586	0.888
29911063	rs199474485	T/G	0.190[3]-0.339[1]	0.328	0.438/0.109	**0.380**	p.I121R	missense variant	Not Reported	0.000931	0.894
29910577	rs45617033	C/T	0[5]-0.00218[2]	5.9004e-05[1]	-	**0.0540**	p.P39P	synonymous variant	Not Reported	0.00205	0.759
29912188	rs9260184	T/C	0.351[2]-0.370[1]	0.399	-	0.478		intron variant	Not Reported	0.00212	0.904
29911019	rs9260149	T/C	0.241-0.375[1]	0.398	0.484/0.156	0.458		intron variant	Not Reported	0.00221	0.902
29911037	rs9260152	G/C	0.144[2]-0.391[1]	0.414	0.453/0.188	0.483		splice region variant/intron variant	Not Reported	0.00234	0.903
29911307	rs199474578	G/A	0.019[2]-0.014[1]	0.023	-	**0.0499**	p.T202T	synonymous variant	Not Reported	0.00255	1.27
29910663	rs707910	G/A	0.091[3]-0.187[5]	0.047	0.906/0.094	**0.0544**	p.R68K	missense variant	Not Reported	0.00265	1.24
29910508	rs9260129	A/G	0.313[3]-0.384[1]	0.750	0.531/0.438	0.253		intron variant	Not Reported	0.00265	0.889
29910892	rs9260145	A/G	0.376[4]-0.383[5]	0.405	-	**0.481**		intron variant	Not Reported	0.00285	0.906

29910437	rs9260124	C/T	0.344[6]-0.376[1]	0.449	-	**0.462**		intron variant	Not Reported	0.00293	0.904
29911092	rs1136702	G/T	0.163[2]-0.270[1]	0.250	0.375-0.062	**0.327**	p.G131W	missense variant	Not Reported	0.00335	0.901
29909499	rs71554994	A/T	-	-	-	0.281		intron variant	Not Reported	0.00348	0.897
29910730	rs576213756	T/ TCTCCAAGACCAA	0.001[3]	0.008	-	**0.483**	p.N90NLQDQ	inframe insertion	Not Reported	0.00354	0.908
29909021	rs2571420	C/G	0.090[3]-0.188[5]	0.047	0.906/0.094	**0.0642**		upstream gene variant	Not Reported	0.00375	1.21
29910034	rs2735114	G/A	0.387[1]-0.398[3]	0.414	0.516/0.156	**0.498**		5' UTR variant	Not Reported	0.00394	0.911
29911154	rs1059509	C/A	0.447[1]-0.458[3]	0.523	0.516/0.266	0.384	p.N151K	missense variant	Not Reported	0.00438	0.907
29911727	rs9280790	T/C	-	-	-	0.490		intron variant	Not Reported	0.00442	0.904

*Allelic frequencies were estimated using 1000G (*1000 genomes project phase 3)* data from Mexican American from Los Angeles population and Latino population from *GnomAD* database. Genotype frequencies also were estimated using *1000G.* Average frequency was consulted from highest and lowest frequencies from *GnomAD,*[1] *ExAC,*[2] *1000G,*[3] *ALSPAC,*[4] *TWINSUK*[5] and *GO-ESP*[6] information published in *dbSNP* database, available in https://www.ncbi.nlm.nih.gov/snp/

Table S2. Variants found in HLA-B loci related to type 2 diabetes in mexican population with statistical significance p < 0.005.

**Position in chromosome 6 (GRCh37/hg19)**	**dbSNP ID**	**Reference/effect allele**	**Effect allele average frequency**	**Effect allelic frequency in Mexican population***	**Effect genotype frequency hetero/ homozygote***	**Protein change**	**Consequence**	**Clinical significance (Other phenotype, diseases or trait related in *ClinVar*)**	***P* Value**	**Odds ratio**
31324100	rs1050654	G/T	0.413[2]	0.469	-	p.R155S	missense variant	Atopic dermatitis	0.000103	0.868
31323353		G/ GTGTCTTTGGGGGGTCACA		-	-	p.H212HVTPQRH	inframe insertion	Not Reported	0.000978	0.901
31324086	rs709054	G/A	0.016[3]	0.008	0.016/-	p.A159A	synonymous variant	Not Reported	0.00152	0.891
31323233	rs709052	A/G	0.436[1]-0.416[5]	0.484	0.594/0.182	p.T252T	synonymous variant	Not Reported	0.00167	0.904
31323885	rs41549217	G/T	0.031-0.0520	0.055	0.891/0.109		intron variant	Clozapine-induced agranulocytosis	0.00285	0.869
31324077	rs709053	C/G	0.393[3]	0.461	0.764/0.094	p.T162T	synonymous variant	Not Reported	0.00314	0.898
31323567	rs4081560	G/A	0.141[1]-0.090[3]	0.211	0.391/0.016		intron variant	Not Reported	0.00376	0.895
31323677	rs4081559	C/T	0.090[3]-0.1154[1]	0.211	0.391/0.016		intron variant	Not Reported	0.00434	0.897
31323875	rs41560220	C/T	0.078[1]-0.054[3]	0.141	0.250/0.016		intron variant	Not Reported	0.00439	0.888

*Allelic frequencies were estimated using 1000G (*1000 genomes project phase 3)* data from Mexican American from Los Angeles population and Latino population from *GnomAD* database. Genotype frequencies also were estimated using *1000G.* Average frequency was consulted from highest and lowest frequencies from *GnomAD,*[1] *ExAC,*[2] *1000G,*[3] *ALSPAC,*[4] *TWINSUK*[5] and *GO-ESP*[6] information published in *dbSNP* database, available in https://www.ncbi.nlm.nih.gov/snp/

Table S3. Variants found in HLA-C loci related to type 2 diabetes in mexican population with statistical significance p < 0.005.

Position in chromosome 6 (GRCh37/hg19)	dbSNP ID	Reference/effect allele	Effect allele average frequency	Effect allele frequency in Mexican population*	Effect genotype frequency hetero/ homozygote*	Protein change	Consequence	Clinical significance (Other phenotype, diseases or trait related in *ClinVar*)	*P* Value	Odds ratio
31239407	rs17408553	G/T	0.352[6]-0.433[4]	0.344	0.500/0.094	p.N104K	missense variant	Not Reported	0.00203	1.11
31239417	rs2308557	C/T	0.337[5]-0.4021	0.344	0.500/0.094	p.S101N	missense variant	Not Reported	0.00212	1.11
31239101	rs1131115**	G/T	0.337[5]-0.434[6]	0.625	0.112/0.354	p.S123Y	missense variant	Not Reported	0.00254	1.14
31236998	rs2001181	C/T	0.111[3]-0.139[1]	0.906	0.188/0.812	p.N104K	intron variant	Not Reported	0.00265	1.14
31236853	rs1065711	G/A	0.111[3]-0.155[4]	0.906	0.188/0.812	p.S101N	3' UTR variant	Not Reported	0.00266	1.14
31239306	rs7383157	T/G	0.111[3]-0.129[1]	0.906	0.188/0.812	p.S123Y	intron variant	Not Reported	0.00375	1.13

*Allelic frequencies were estimated using 1000G (1*000 genomes project phase 3)* data from Mexican American from Los Angeles population and Latino population from *GnomAD* database. Genotype frequencies also were estimated using *1000G.* ** This variant was found in *GWAS SIGMA* and *SIGMA exome chip analysis.* Average frequency was consulted from highest and lowest frequencies from *GnomAD,*[1] *ExAC,*[2] *1000G,*[3] *ALSPAC,*[4] *TWINSUK*[5] and *GO-ESP*[6] information published in *dbSNP* database, available in https://www.ncbi.nlm.nih.gov/snp/.

Table S4. HLA-A*03:01:01:01 protein sequence variants found in mexican population with type 2 diabetes.

**Amino acid residue position**	**Reference**	**Variants registered IPD-IMGT/HLA database**			**T2D GWAS SIGMA**
	**Amino acid Codon**	**Amino acid**	**Codon**	**Database number of registers**	**Amino acid Codon**
68	Arginine AGG	Lysine	AAG	4968	Lysine AAG
		Lysine	AAA	4	
		Arginine	AGG	3	
		Glutamic Acid	GAG	2	
202	Threonine ACG	Arginine	AGG	2381	Threonine ACA
		Arginine	AGA	410	
		Lysine	AAA	1	
		Tryptophan	TGG	1	
		Arginine	CGA	1	
		Serine	AGT	1	
		Lysine	AAG	1	

The HLA polymorphisms register was obtained from IPD-IMGT/HLA database using the *polymorphism search tool* available at https://www.ebi.ac.uk/ipd/imgt/hla/polymorph.html.

Table S5. HLA-C*01:02:01:01 protein sequence variants found in mexican population with type 2 diabetes.

Amino acid residue position	Reference Amino acid Codon	Variants registered IPD-IMGT/HLA database			T2D GWAS SIGMA Amino acid Codon
		Amino acid	Codon	Database number of registers	
101	Serine TCG	Cysteine	TGC	4885	Asparagine TTG*
		Cysteine	TGT	4	
		Tyrosine	TAC	4	
		Tryptophan	TGG	3	
		Serine	AGC	2	
		Glycine	GGC	2	
		Arginine	CGC	1	
104	Asparagine TTG	Glycine	GGG	4871	Lysine TTC*
		Glycine	GGT	8	
		Glutamic Acid	GAG	6	
		Arginine	AGG	4	
		Glycine	GGA	4	
		Arginine	CGG	3	
		Alanine	GCG	3	
		Tryptophan	TGG	1	
		Valine	GTG	1	
123	Serine AGA	Tyrosine	ATG	4893	Tyrosine ATA
		Tyrosine	ATA	4	
		Aspartic Acid	GAC	2	
		Phenylalanine	TTC	1	
		Histidine	CAC	1	

*Variant not described in the IPD-IMGT/HLA database.

HLA allele polymorphisms were obtained from IPD-IMGT/HLA database, using the *polymorphism search tool* available at https://www.ebi.ac.uk/ipd/imgt/hla/polymorph.html.

Table S6. Polymorphisms found in HLA-A*03:01:01:01 and HLA-C*01:02:01:01 alleles. The residues 26 to 114 correspond to alpha-1 chain and 115 to 204 to alpha-2 chain; both part of the hypervariable region in the peptide-binding cleft related with antigen recognition. Variants found in our analysis are marked with a red arrow. The position references were taken from https://www.uniprot.org/uniprot/P01891**.** HLA allele polymorphisms were obtained from IPD-IMGT/HLA database, using the polymorphism search tool available at https://www.ebi.ac.uk/ipd/imgt/hla/polymorph.html. Only variants with statistical significance less than 0.05 and odds ratio more than 1.0 were considered.

HLA-A*03:01:01:01

Residues

	26	27	28	29	30	31	32	33	34	35	36	37	38	39	40	41	42	43	44	45	46
**VariabIlty**	0	0.1	0	0	0	0.1	0	1.8	0	0	0.1	0	0	0	0	0.3	0	0	0	0	0
**Reference residue**	S	H	S	M	R	Y	F	F	T	S	V	S	R	P	G	R	G	E	P	R	F
**Polymorphic residue**	S	H	S	M	R	Y	F	F	T	S	V	S	R	P	G	R	G	E	P	R	F

	47	48	49	50	51	52	53	54	55	56	57	58	59	60	61	62	63	64	65	66	67
**VariabIlty**	0	0	0	0	0	0	0	0	0.1	0	0	0	0	0	0	0	0	0	0	0	0.2
**Reference residue**	I	A	V	G	Y	V	D	D	T	Q	F	V	R	F	D	S	D	A	A	S	Q
**Polymorphic residue**	I	A	V	G	Y	V	D	D	T	Q	F	V	R	F	D	S	D	A	A	S	Q

	68	69	70	71	72	73	74	75	76	77	78	79	80	81	82	83	84	85	86	87	88
**VariabIlty**	0.3	0	0	0	0	0	0	0	0	0	0	0	0.5	0	0	0	0	0	2.1	1	0
**Reference residue**	R	M	E	P	R	A	P	W	I	E	Q	E	G	P	E	Y	W	D	G	E	T
**Polymorphic residue**	K	M	E	P	R	A	P	W	I	E	Q	E	G	P	E	Y	W	D	G	E	T

	89	90	91	92	93	94	95	96	97	98	99	100	101	102	103	104	105	106	107	108	109
**VariabIlty**	0.7	1.1	0.3	0	0	0.8	0	0	0.4	0.9	0	1.4	1.2	0	0.8	0.8	0.8	0.8	0.8	0	0
**Reference residue**	R	K	V	K	A	H	S	Q	T	H	R	V	D	L	G	T	L	R	G	Y	Y
**Polymorphic residue**	R	N	V	K	A	Q	S	Q	T	D	R	V	D	L	G	T	L	R	G	Y	Y

Residues

	110	111	112	113	114	115	116	117	118	119	120	121	122	123	124	125	126	127	128	129	130
**VariabilIty**	0	0	0	0	0.8	0	0	0	0	1.4	0	1.5	0	0.8	0	0	0	0	0	0.9	0
**Reference residue**	N	Q	S	E	A	G	S	H	T	V	Q	R	M	Y	G	C	D	V	G	S	D
**Polymorphic residue**	N	Q	S	E	A	G	S	H	T	**I**	Q	**I**	M	Y	G	C	D	V	G	S	D

	131	132	133	134	135	136	137	138	139	140	141	142	143	144	145	146	147	148	149	150	151
**VariabilIty**	0.8	0	0.4	0	0	0	0	1.8	0	1.4	0	0	0	0	0	0	0	0	0	0	1
**Reference residue**	W	R	F	L	R	G	Y	H	Q	Y	A	Y	D	G	K	D	Y	I	A	L	K
**Polymorphic residue**	**G**	R	F	L	R	G	Y	**R**	Q	**D**	A	Y	D	G	K	D	Y	I	A	L	**N**

	152	153	154	155	156	157	158	159	160	161	162	163	164	165	166	167	168	169	170	171	172
**VariabilIty**	0	0	0	0	0	0	0	0	0	0	0.1	0	0	0	0.9	0	1	0.9	0	0	0
**Reference residue**	E	D	L	R	S	W	T	A	A	D	M	A	A	Q	T	T	K	H	K	W	E
**Polymorphic residue**	E	D	L	R	S	W	T	A	A	D	M	A	A	Q	**I**	T	K	**R**	K	W	E

	173	174	175	176	177	178	179	180	181	182	183	184	185	186	187	188	189	190	191	192	193
**VariabilIty**	0.6	0.3	0.8	1.4	0	0	0	1.7	0	0.3	0	0	0.4	0	0.9	0	0	0.7	0.7	0	0
**Reference residue**	A	A	H	V	A	E	Q	L	R	A	Y	L	E	G	T	C	V	E	W	L	R
**Polymorphic residue**	A	A	H	**E**	A	E	Q	L	R	A	Y	L	**D**	G	T	C	V	E	W	L	R

	194	195	196	197	198	199	200	201	202	203	204
**VariabilIty**	0	0.2	0	0	0	0	0	0	0	0	0
**Reference residue**	R	Y	L	E	N	G	K	E	T	L	Q
**Polymorphic residue**	R	Y	L	E	N	G	K	E	T	L	Q

HLA-C*01:02:01:01

Residues

	26	27	28	29	30	31	32	33	34	35	36	37	38	39	40	41	42	43	44	45	46
**VariabilIty**	0	0	0	0	0.3	0.1	0	1.5	0	0.6	0	0	0.3	0	0.4	0.1	0	0	0	0.9	0
**Reference residue**	S	H	S	M	R	Y	F	Y	T	A	V	S	R	P	G	R	G	E	P	R	F
**Polymorphic residue**	S	H	S	M	K	Y	F	F	T	S	V	S	R	P	G	R	G	E	P	R	F

	47	48	49	50	51	52	53	54	55	56	57	58	59	60	61	62	63	64	65	66	67
**VariabilIty**	0	0.9	0	0	0	0.1	0	0	0	0	0	0	0.7	0	0	0	0	0	0	0	0
**Reference residue**	I	A	V	G	Y	V	D	D	T	Q	F	V	R	F	D	S	D	A	A	S	P
**Polymorphic residue**	I	S	V	G	Y	V	D	D	T	Q	F	V	R	F	D	S	D	A	A	S	P

	68	69	70	71	72	73	74	75	76	77	78	79	80	81	82	83	84	85	86	87	88
**VariabilIty**	0	0	0	0	0	0.2	0	0	0	0	0.1	0	0	0	0	0	0	0	0	0	0
**Reference residue**	R	G	E	P	R	A	P	W	V	E	Q	E	G	P	E	Y	W	D	R	E	T
**Polymorphic residue**	R	G	E	P	R	A	P	W	V	E	Q	E	G	P	E	Y	W	D	R	E	T

	89	90	91	92	93	94	95	96	97	98	99	100	101	102	103	104	105	106	107	108	109
**VariabilIty**	0	0.6	0	0	0	0	0	0	1	0	0	0	1.1	0	0	1	0	0	0	0	0
**Reference residue**	Q	N	Y	K	R	Q	A	Q	A	D	R	V	S	L	R	N	L	R	G	Y	Y
**Polymorphic residue**	Q	K	Y	K	R	Q	A	Q	T	D	R	V	N	L	R	K	L	R	G	Y	Y

	110	111	112	113	114	115	116	117	118	119	120	121	122	123	124	125	126	127	128	129	130
**VariabilIty**	0	0	0	0	0.9	0.2	0	0	0.8	1	0	1	0	1.1	0	0	0	0.5	0	0	0
**Reference residue**	N	Q	S	E	A	G	S	H	T	L	Q	R	M	S	G	C	D	L	G	P	D
**Polymorphic residue**	N	Q	S	E	A	G	S	H	T	L	Q	W	M	Y	G	C	D	L	G	P	D

	131	132	133	134	135	136	137	138	139	140	141	142	143	144	145	146	147	148	149	150	151
**VariabilIty**	0	0	0	0	0	0	0.4	1	0	1.7	0	0	0	0.1	0	0	0	0	0	0	0
**Reference residue**	G	R	L	L	R	G	Y	D	Q	S	A	Y	D	G	K	D	Y	I	A	L	N
**Polymorphic residue**	G	R	L	L	R	G	Y	D	Q	Y	A	Y	D	G	K	D	Y	I	A	L	N

Residues

	152	153	154	155	156	157	158	159	160	161	162	163	164	165	166	167	168	169	170	171	172
**VariabilIty**	0	0	0	0	0	0	0	0	0	0	0.5	0	0	0	0	0.2	0	0	0	0.7	0
**Reference residue**	E	D	L	R	S	W	T	A	A	D	T	A	A	Q	I	T	Q	R	K	W	E
**Polymorphic residue**	E	D	L	R	S	W	T	A	A	D	T	A	A	Q	I	T	Q	R	K	W	E

	173	174	175	176	177	178	179	180	181	182	183	184	185	186	187	188	189	190	191	192	193
**VariabilIty**	0	0	0	1.1	0	0	0	1.7	0	0	0	0	0	0	1.2	0	0	0	0	0	0
**Reference residue**	A	A	R	E	A	E	Q	W	R	A	Y	L	E	G	T	C	V	E	W	L	A
**Polymorphic residue**	A	A	R	E	A	E	Q	R	R	A	Y	L	E	G	T	C	V	E	W	L	A

	194	195	196	197	198	199	200	201	202	203	204
**VariabilIty**	0.2	0	0	0.6	0	0.2	0.1	0.7	0	0	0
**Reference residue**	R	Y	L	E	N	G	K	E	T	L	Q
**Polymorphic residue**	R	Y	L	E	N	G	K	E	T	L	Q

Figure S1. Methods flowchart.

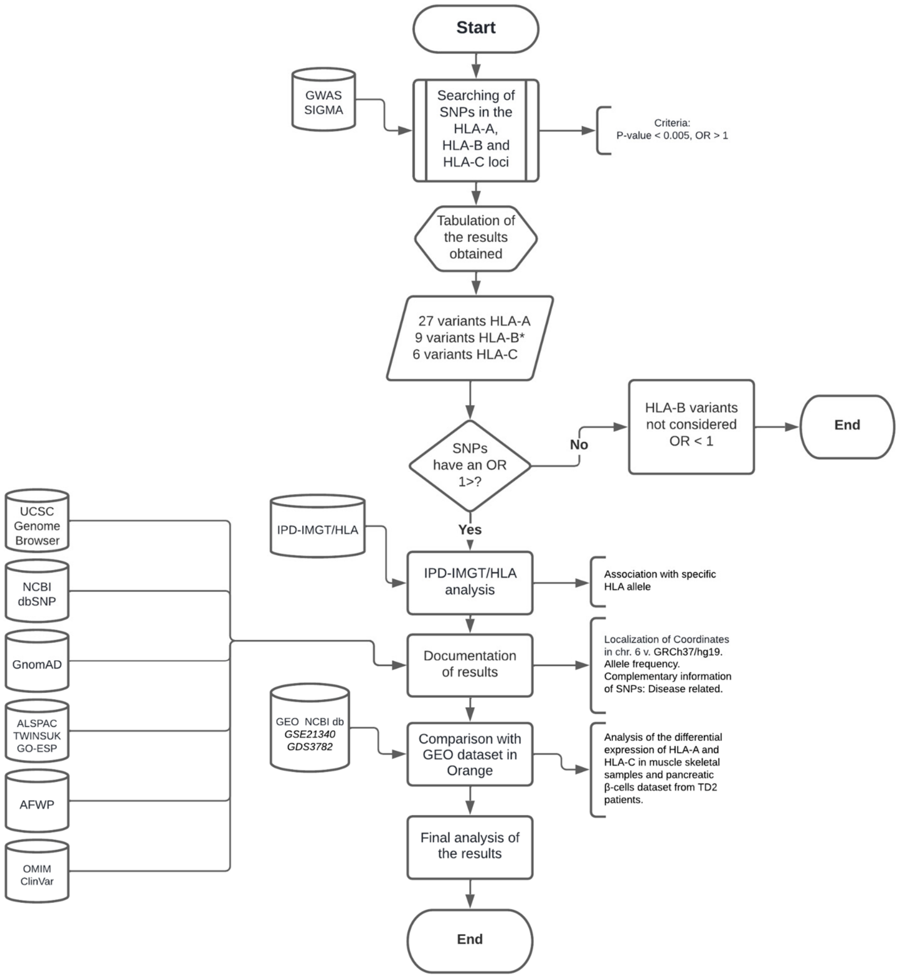

Figure S2. HLA-A*03:01:01:01 allele frequency in worldwide populations.

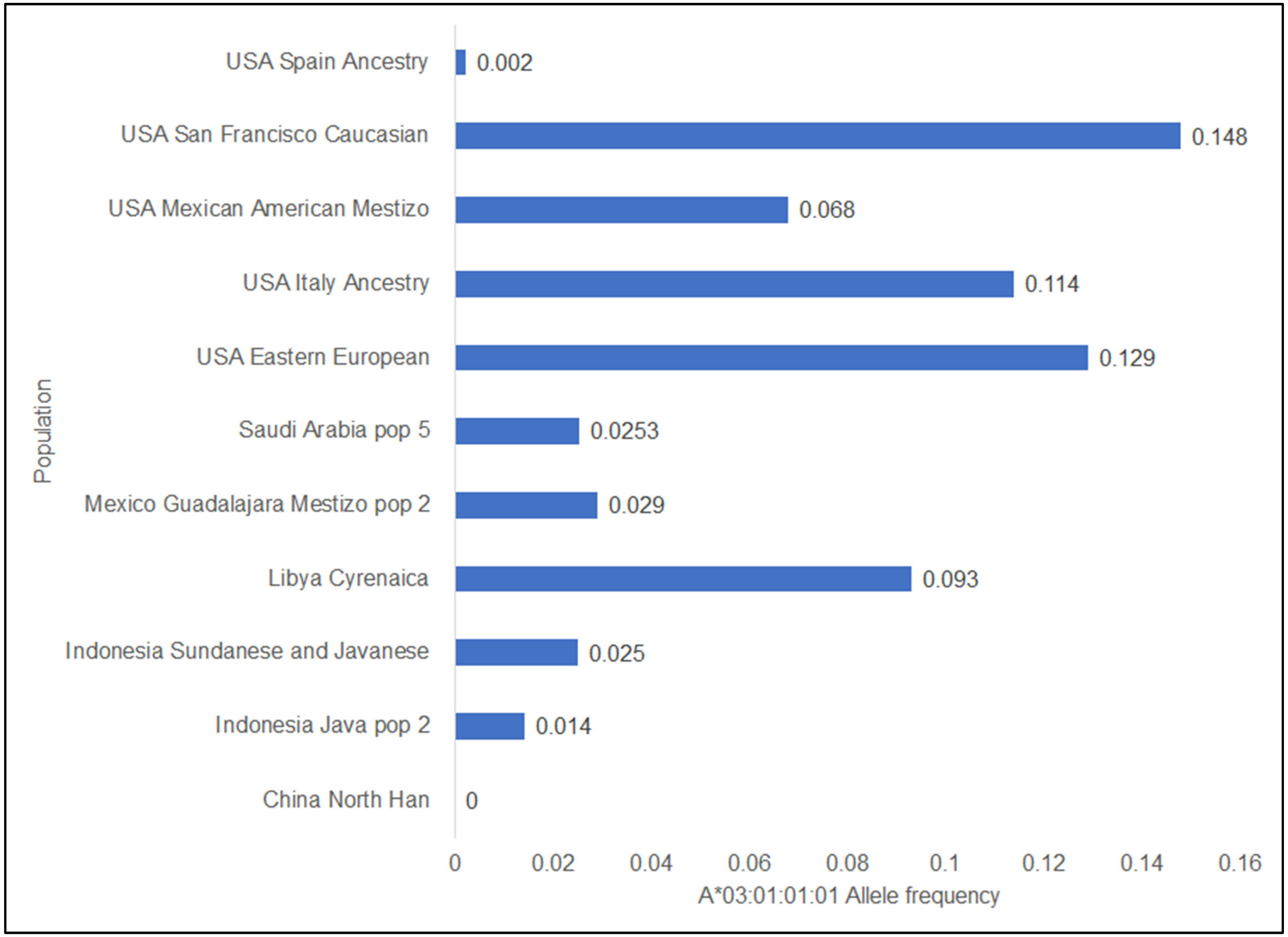

For the allele HLA-A*03:01:01:01, the group that presented the highest frequency was the USA San Francisco Caucasian population (0.148). Frequencies obtained from *Allele frequencies in Worldwide populations* database, available at http://www.allelefrequencies.net/.

Figure S3. HLA-C*01:02:01:01 frequency in worldwide populations.

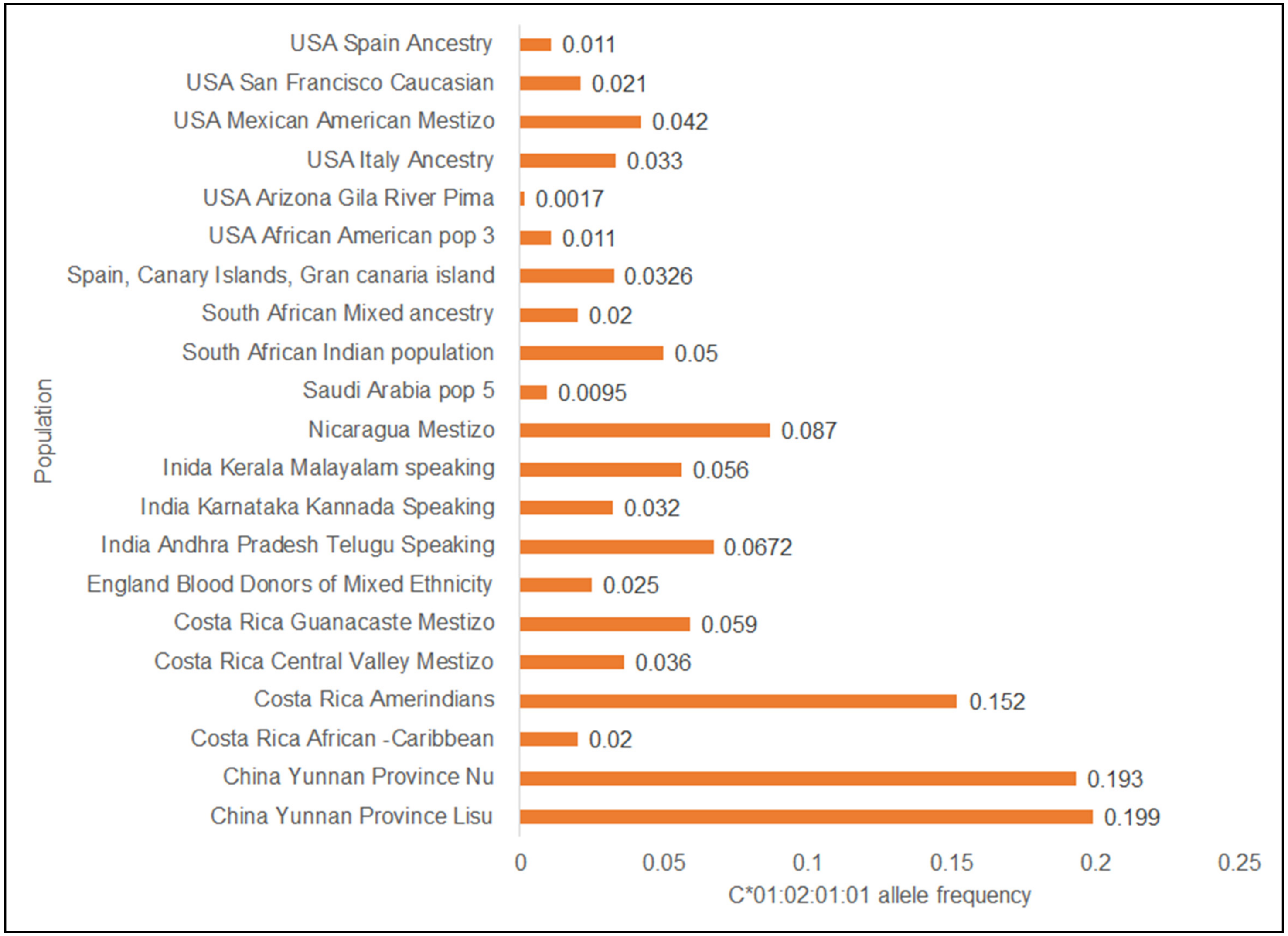

For the allele HLA-C*01:02:01:01, the group that presented the highest frequency was the China Yunnan Province Lisu population (0.199). Frequencies obtained from *Allele frequencies in Worldwide populations* database, available at http://www.allelefrequencies.net/.

Figure S4. HLA.A and HLA-C differential expression values from muscle tissue obtained from a group with T2D compared to a control group with a positive family history for T2D and a control group with a negative family history for diabetes.

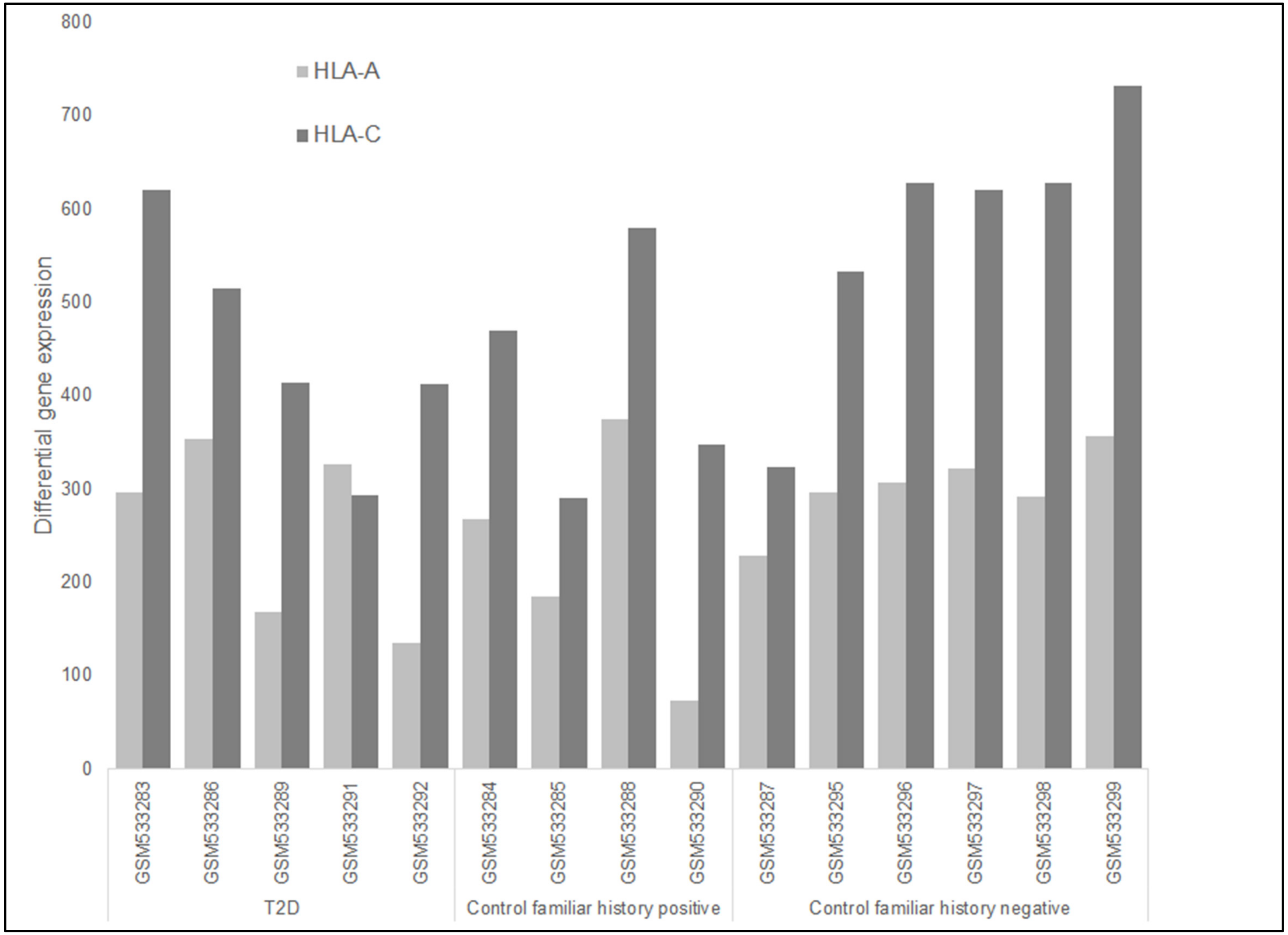

No significant differences were found between the different groups using the Mann Whitey U test when compared individually, but in a group there are differences. Genetic expression data obtained from the database *Omnibus (GEO)* (https://www.ncbi.nlm.nih.gov/gds) with access ID GSE21340 *Human skeletal muscle - type 2 diabetes and family history positive individuals - Mexican American*.

Figure S5. Fisher's exact test of the expression values obtained from the group with Type 2 Diabetes (DT2) compared to a control group with a positive family history for DT2 and a control group with a negative family history for diabetes.

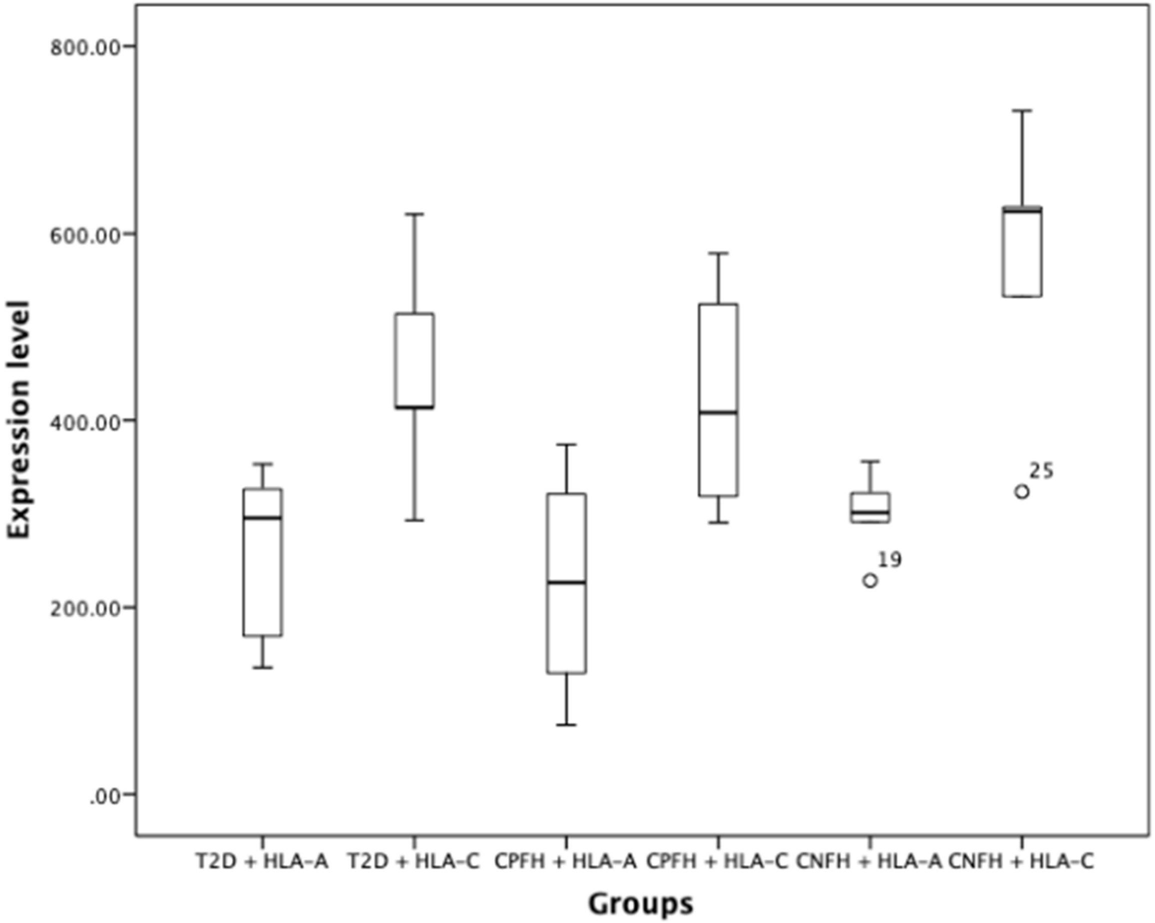

The differences between the levels of expression between HLA A ($p < 0.027$) HLA C ($p < 0.047$) of the group with T2D and the control group with negative family history are the most important in our analysis, while there are no differences between the group with T2D and the control group with positive family history. Genetic expression data obtained from the database *Omnibus (GEO)* (https://www.ncbi.nlm.nih.gov/gds) with access ID GSE21340 *Human skeletal muscle - type 2 diabetes and family history positive individuals - Mexican American*.
